# Identification of Cell Surface Molecules That Determine the Macrophage Activation Threshold Associated With an Early Stage of Malignant Transformation

**DOI:** 10.3389/fimmu.2021.749597

**Published:** 2021-10-12

**Authors:** Camille Jacqueline, Matthew Dracz, Sarah Boothman, Jonathan S. Minden, Rachel A. Gottschalk, Olivera J. Finn

**Affiliations:** ^1^ Department of Immunology, University of Pittsburgh, Pittsburgh, PA, United States; ^2^ Department of Biological Sciences, Carnegie Mellon University, Pittsburgh, PA, United States

**Keywords:** breast cancer, TNF-α, 2D-DIGE, phagocytosis, annexin A1, CEACAM1

## Abstract

The ability of immune cells to sense changes associated with malignant transformation as early as possible is likely to be important for the successful outcome of cancer immunosurveillance. In this process, the immune system faces a trade-off between elimination of cells harboring premalignant or malignant changes, and autoimmune pathologies. We hypothesized that the immune system has therefore evolved a threshold for the stage of transformation from normal to fully malignant cells that first provides a threat (danger) signal requiring a response. We co-cultured human macrophages with a unique set of genetically related human cell lines that recapitulate successive stages in breast cancer development: MCF10A (immortalized, normal); MCFNeoT (benign hyperplasia); MCFT1 (atypical hyperplasia); MCFCA1 (invasive cancer). Using cytokines-based assays, we found that macrophages were inert towards MCF10A and MCFNeoT but were strongly activated by MCFT1 and MCFCA1 to produce inflammatory cytokines, placing the threshold for recognition between two premalignant stages, the earlier stage MCFNeoT and the more advanced MCFT1. The cytokine activation threshold paralleled the threshold for enhanced phagocytosis. Using proteomic and transcriptomic approaches, we identified surface molecules, some of which are well-known tumor-associated antigens, that were absent or expressed at low levels in MCF10A and MCFNeoT but turned on or over-expressed in MCFT1 and MCFCA1. Adding antibodies specific for two of these molecules, Annexin-A1 and CEACAM1, inhibited macrophage activation, supporting their role as cancer “danger signals” recognized by macrophages.

## Introduction

Medical and evolutionary sciences have traditionally developed in relative isolation (1). In the 1970s, evolutionary and ecological concepts began to be applied to cancer initiation and progression (2, 3). Since this seminal work, several studies explored the processes of somatic cellular selection and evolution leading to malignant transformation, metastasis or resistance to therapies (4–6). In addition to the evolution of clonal heterogeneity inside the tumor, tumors also evolve in complex and multifaceted ecological contexts. Indeed, tumors are composed of mixtures of cancer cells and non-cancer cells, which compose the tumor microenvironment (TME). Tumor cells vary in their ability to evade the immune system and the “invisible” clones present a selective advantage and proliferate at the expense of others (7). It is now accepted that this co-evolutionary process, called immunoediting, is ongoing in most cancers and involves the immune response as well as immune evasion by some tumor cells (8). Application of evolutionary biology to the understanding of the crosstalk between cancer and immune cells is a promising strategy to better understand the bases of cancer vulnerability. The need for self-tolerance to avoid auto-immunity and the need to eliminate cancer that arises from self, may have exerted a strong selective pressure on the evolution of cancer immunosurveillance (9, 10). Unlike viruses or bacteria, premalignant cells may be particularly challenging for the immune system because they are mostly self with initially only a few characteristics of tumor cells (11).

Macrophages play an important role in the initiation of immune responses that eventually lead to adaptive immunity and immune memory. Their ability to sense “danger signals” on cells undergoing malignant transformation, similarly to how they sense danger signals from pathogens, may determine if and when cancer immunosurveillance is initiated. In the context of microbial stimuli, an evolutionarily conserved threshold for “danger discrimination” controls inflammatory cytokines production (12); MAPK were activated above a certain concentration of microbial products, setting an inflammatory activation threshold in both mouse and human macrophages. Recently, it was shown that in squamous cell carcinoma of the lung, innate inflammatory responses are low in benign lesions but increase with higher grade pre-invasive lesions, suggesting the existence of a threshold of activation of innate immune responses to cancer (13).

We tested this hypothesis in a unique set of human breast cell lines derived on the same genetic background, that recapitulate several steps in breast cancer progression: MCF10A cell line, immortalized but not transformed; MCFNeoT and MCFT1 cell lines, H-ras transformed, corresponding to premalignant hyperplasia and atypical hyperplasia, respectively; and MCFCA1, fully malignant invasive tumor cell line (14, 15). We exposed macrophages to these cells and assessed their activation by the secretion of several cytokines. Macrophages produced IL-10 upon encounter with MCF10A and MCFNeoT but switched to TNF-α and IL-1β when co-incubated with hyperplastic MCFT1 and fully transformed MCFCA1, suggesting an activation threshold at the premalignant stage represented by MCFT1. We also found that increased phagocytic activity followed the same threshold with macrophages forming conjugates with MCF10A and MCFNeoT but fully engulfing MCFT1 and MCFCA1. We measured differences in the transcriptome and the proteome between these cell lines and found several candidate molecules whose expression correlates with the threshold for macrophage activation. We found expression of the same molecules and macrophage infiltration in early stages of malignant transformation in breast tissue samples, recapitulating our findings *in vitro*.

## Materials and Methods

### Antibodies

Mouse monoclonal antibody 4H5, gift from the late Dr. Hilgers (Free University, Amsterdam), was used to stain the hypoglycosylated form of MUC1. Her-2/neu was detected with Herceptin ^®^ (Trastuzumab, Genentech Inc., San Francisco, CA, USA) and CEACAM1 with antibody CEACAM1/CD66a (R&D Systems, Minneapolis, MN, USA). Anti-Serpin B1 (clone 3B4) antibody was obtained from Novus Biologicals (Centennial, CO, USA). Anti-Annexin A1 clone EPR19342 and anti-CD68 (EPR20545) antibodies were purchased from Abcam (Cambridge, UK). Anti-Annexin A1 (74/3) and anti-Calregulin (clone F-4) was purchased from Biolegend (San Diego, CA, USA) and Santa Cruz Biotechnology (Dallas, TX, USA) respectively. Anti-PECAM1 (clone WM59) and FITC-CD47 (clone B6H12) were purchased from BD Biosciences (Franklin Lakes, NJ, USA). APC-conjugated F(ab’)2 fragment specific to human IgG (Jackson Immunoresearch, West Grove, PA, USA) and FITC-conjugated goat anti-mouse IgG (Invitrogen, Carlsbad, CA, USA) were used as secondary antibodies. HRP-conjugated goat anti-rabbit (IgG) and goat anti-mouse (IgG) were purchased from Abcam and Jackson ImmunoResearch respectively.

### Cell Lines

MCF10A cell line was purchased from ATCC (Manassas, VA, USA). MCFNeoT, MCFT1, and MCFCA1 cells were obtained from the Barbara Ann Karmanos Cancer Institute (Detroit, MI). The four cell lines were maintained as monolayers in Dulbecco’s Modified Eagle’s Medium-F12 (DMEM/F12) (Gibco, 11320033) supplemented with 5% horse serum (Gibco, 16050122), 1% penicillin/streptomycin (Lonza, 17-602E), 0.5 μg/ml hydrocortisone (StemCell, 37150), 100 ng/ml cholera toxin (Sigma, C-8052), 10 μg/ml insulin (Gibco, 1285014), and 20 ng/ml recombinant human EGF (Invitrogen, PHG0311). THP-1 monocyte cell line was purchased from ATCC (TIB-202, Manassas, VI) and TNF-reporter cell line (THP1-B5) was a gift from Dr. Ian Fraser (NIH/NIAID). Cells were cultured in RPMI-1640 (Life Technologies, Carslbad, CA) supplemented with 10% FBS, 1% Penicillin/Streptomycin and 1% Sodium Pyruvate. MCF cells were trypsinized off the plates. All cell lines were regularly tested for Mycoplasma contamination by PCR.

### Macrophage Generation

Human peripheral blood mononuclear cells (PBMCs) were isolated from buffy coats of healthy blood donors (purchased from Vitalant, Pittsburgh, PA) by FicollTM (Sigma-Aldrich) density gradient. Monocytes were sorted by magnetic-activated cell sorting (MACS) using magnetic beads conjugated with anti-human CD14 (CD14 MicroBeads, human, Miltenyi Biotech, Bergish Gladbach, Germany) and cultured for 5 days in RPMI 1640 culture medium and M-CSF (100 ng/mL; R&D systems) to differentiate them into non-polarized (M0) monocyte-derived macrophages. Macrophages were generated from THP-1 and THP1-B5 monocyte cell lines by incubation for 48 hours with 100 nM phorbol 12-myristate 13-acetate (PMA, Sigma, P8139), followed by 48 hours incubation in RPMI medium. 5mM EDTA was used to detach macrophages.

### Antibody Blocking of Macrophage Activation and Macrophage Stimulation With Danger Signals

125,000 MCF cell lines were pre-incubated with 1:10 dilution of an mouse IgG1 isotype control antibody (0.5 mg/ml), anti-Serpin B1 (1 mg/ml), anti-Annexin A1 (1 mg/ml) or anti-CEACAM1 (0.5 mg/ml) for 30 min at 4°C before co-incubation with macrophages. Macrophages were stimulated with seven 2-fold serial dilutions of the 50 µg/ml top solution of CEACAM1 and Annexin A1 protein (R&D systems) for 24 hours.

### Co-Cultures

Macrophages were always pretreated with 2% FC receptor binding inhibitor (ThermoFisher) for 15 min at 4°C before co-incubation. Co-incubations were carried out in two different step-ups: i) macrophages were plated simultaneously with MCF cells to a ratio macrophages/MCF cells of 1:5 or 1:10. ii) macrophages were plated in the bottom of the plate and MCF cells in a 96-well 0.4 µm transwell (Corning) with the same 1:5 and 1:10 macrophages/MCF cells ratio. All coincubation were carried out in duplicate in 96-well plates, in 150ul of MCF medium and for 24 hours. When indicated in the legend, MCF cells were treated with neuraminidase (1:1000, Sigma-Aldrich) for 2 hours in low-adherent plates before co-incubation.

### Cytokine-Based Assays (CBA) and ELISA

At the end of the co-incubation supernatants were collected for the determination of cytokine production and were stored at -80°C until used. Bead-based multiplex cytokine assay was used to measure the following cytokines: IL-1β, IFN-α2, IFN-γ, TNF-α, MCP-1, IL-4, IL-6, IL-8, IL-10, IL-12p70, IL-15, IL-17A, Il-18, IL-23, IL-33 (LEGENDplex™, Biolegend, San Diego, CA, USA). A serial dilution of the inflammatory cytokine panel was run on the same plate according to the manufacturer’s instructions and read using a Fortessa flow cytometer. Ten thousand total events were recorded per sample and cytokines were considered undetectable below 2 pg/ml. Alternatively, human TNF-α ELISA Max kit (Biolegend) was used to measure the concentration of TNF-α in the supernatant of stimulated macrophages according to manufacturer’s protocol.

### Phagocytosis Assay

Macrophages and MCF cells were washed twice in PBS, and labeled with 1 µM CellTrace Yellow, Violet or CSFE (ThermoFisher, Waltham, MA, USA) according to manufacturer’s protocol. After 2 hours of co-incubation in 6-well plates, cells were stained with a viability dye (1:1000 dilution in PBS, Ghost Red 780, #13-0865, TONBO Biosciences, San Diego CA, USA) for 15 min at 4°C. Samples were run on a Fortessa flow cytometer and gated based on negative signal for APC-Cy7 (*i.e.*, live cells). AMNIS image cytometry was performed using an Amnis cytometer. Cells were first visualized in a bright field and identified as macrophages (PacBlue) or MCF cells (FITC). The IDEAS software 6.2 was used to evaluate the percentage of doublets using the same gating strategy as for the phagocytosis assay. The images were merged to confirm the uptake of MCF cells by macrophages. The internalization score was measured using the internalization wizard of the IDEAS software.

### Flow Cytometry

Cells were trypsinized, washed, collected and then stained with a viability dye (1:1000 dilution in PBS, Ghost Red 780, #13-0865, TONBO Biosciences, San Diego CA, USA) for 15 min at 4°C. Cells were stained with the anti-MUC1 antibody 4H5 (1:100), anti-HER2 antibody trastuzumab (1:2000), anti-CEACAM1 antibody (1:100), anti-Serpin B1 (1:100), anti-Annexin A1 (1:400), anti-CRT (1:100), anti-PECAM1 (1:100) and anti-CD47 (100) diluted in flow cytometry buffer (PBS + 1% BSA), for 30 min at 4°C followed by two washes with FACS buffer. Cells were then stained with secondary goat anti-mouse IgG or F(ab’)2 anti-human IgG (1:200 dilution in FACS buffer) for 30 min at 4°C. Samples were run on a Fortessa flow cytometer and gated based on negative signal for APC-Cy7 (*i.e.*, live cells). APC (human) or FITC (mouse) mean fluorescence intensities were measured. 30,000 total events were recorded per sample.

### Dual Luciferase Assay of THP1-B5 Cell TNF-α Production

After stimulation, the cells were washed once in PBS and lysed in passive lysis buffer (Promega). Firefly and renilla luciferase activities were determined using SpectraMax i3X and the software Softmax Pro 7.0.3 with an 5s acquisition. The ratio of firefly luminescence to renilla luminescence was used to reflect TNF-α production in response to stimulation.

### Microscopy and 3-D Reconstruction

3-D overlay cultures were generated following the published method (16). Briefly, 8-chamber slides (Falcon CultureSlides, #354118) were coated with 40 µl of Matrigel (Corning ^®^ Matrigel ^®^ Matrix, #356234) and 5000 cells/well were seeded in medium containing 2% Matrigel and 5 ng/ml EGF. After 7-9 days, 3-D cultures in Matrigel were washed twice with media before seeding PMA-treated THP-1 (40,000 cells/well). THP-1 were previously labelled with CellTrace CFSE (Carboxyfluorescein succinimidyl ester) as described above. After 24 hours of incubation, bright-field and fluorescence images were taken using the Z-stack option on an Olympus Fluoview 1000 confocal microscope at the Center for Biologic Imaging, University of Pittsburgh. The pictures were taken under fixed exposure conditions. 3-D reconstruction was performed with NIS-Elements (Nikon Instruments Inc., USA). 3-D cultures were delimited manually on each stack and FITC-positive macrophages were detected using the 3-D spot detection function. The infiltration score was determined as the number of macrophages detected inside the spheroid divided by the total volume of the spheroid, multiplied by 1,000,000.

### 2D-DIGE and Liquid Chromatography/Mass Spectrometry (LC/MS) Analysis

Total cell lysates were generated from a confluent 10 cm^2^ culture plate by scraping the cells with 100 µl of lysis buffer (7 M Urea, 2 M Thiourea, 10 mM Hepes pH 8.0, 10 mM DTT, 4% CHAPS) followed by 30 min incubation on ice, 7 cycles of 30 s ON/30s OFF sonicator and centrifugation for 15 min at 14,000 rpm. Extracted protein were stored at -70°C. One hundred µg of untreated and treated samples were labelled with Cy3- and Cy5-NHS minimal-labeling DIGE dyes (GE Healthcare, Uppsala, Sweden) diluted in Dimethylformamide (DMF) (Sigma) for 30 min on ice. Labeling of the two samples was reversed (reciprocal labeling) and run concurrently on a second 2D-DIGE gel to eliminate dye-dependent differences, constituting a technical replicate. First-dimension Isoelectric Point Focusing (IEF), and second-dimension SDS-PAGE were conducted as described (17) with the following modifications. Proteins were separated in the first-dimension on 18 cm pH 3-10NL IPG strips on a Protean i12 IEF Cell apparatus (Bio-Rad) for 32,000 Volt-hours. The samples were then separated on the second-dimension SDS-PAGE in 12% polyacrylamide gels in standard Tris-Glycine-SDS running buffer. After electrophoresis, the gels were fixed in a solution of 40% methanol and 10% acetic acid. The gels were imaged on a custom-built, fluorescent gel imager that housed a robotic spot-cutting head. The resultant fluorescent images were analyzed and selected spots that were then cut from the gels and identified *via* Nano LC-ESI-MS/MS, as described (18). We analyzed 2 biological replicates for each cell lines and 2 technical replicates for each biological replicate. After identification, the characteristics of the proteins and their sequences were obtained through the Uniprot database (https://www.uniprot.org). Finally, we applied Source Extractor to quantify the changes in 2D gels. Source extractor is a neural-network based star/galaxy classifier run by Docker. Once the intensity of each spot extracted, we created a cy3/cy5 ratio and normalized it by the mean intensity of 5 guiding spots. Guiding spots were defined as spots equally expressed in both cell lines (appearing yellow in the gel). The ratios were then log transformed to help with visualization.

### Gene Expression Profiling

Total RNA was isolated from the four MCF cell lines using microKit (Qiagen). The epithelial cell gene profile was examined using nCounter Human Immunology Panel v2 (NanoString Technologies, Seattle, WA, USA). The protocol was carried out at the Genomics Research Core (University of Pittsburgh) using 100 ng of total RNA from each sample following their commercial protocol. Data were analyzed using the NSolver 4.0 software, following the procedure described in the package instructions (19). Normalization of mRNA content, which adjusts for positive control size factors, background noise and housekeeping genes size factors, as well as differential expression, was performed.

### Immunohistochemistry

Human tissue arrays were obtained from BioChain (Newark, CA, USA) and contained 18 cases of normal, premalignant, and malignant breast tissues (#Z7020010). DCIS slides were provided by Dr. Rohit Bhargava (Department of Pathology, UPMC, Pittsburgh). Slides were deparaffinized by baking overnight at 59°C. Endogenous peroxidase activity was eliminated by treatment with 30% H_2_O_2_ for 15 min at room temperature. Antigen retrieval was performed by microwave heating in 0.1% citrate buffer for 10 min. Non-specific binding sites were blocked with 1% BSA. Reaction with anti-CD68 (1:100), anti-CEACAM1 (1:50) and anti-Annexin A1 (1:100) was performed for 1 hour at room temperature. Secondary antibodies were added at 1:100 dilution for 30 min. Positive signals were visualized by a DAB Substrate Kit (cat. #550880, BD Pharmingen) according to the manufacturer’s protocol. Histology sections were viewed on an Olympus BX40 microscope. Images were acquired using Leica DFC420 camera and Leica Application Suite version 2.7.1 R1.

### Statistical Analysis

Significance analyses were performed by using GraphPad Prism software version 7.0 (GraphPad Inc. San Diego, CA). Results were represented as means ± standard error of the mean (SEM) as specified in the legend. Statistical means and significance were analyzed using multiple comparison tests (One way ANOVA). Significance for all experiments was defined as follows: * p<0.05, ** p<0.01, *** p<0.001, **** p<0.0001.

## Results

### Macrophages Exhibit an Activation Threshold in Response to Different Stages of Malignant Transformation

**Figure 1:**
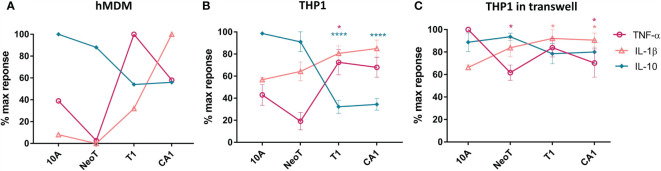
The threshold of macrophage activation occurs between benign hyperplasia and atypical hyperplasia. **(A)** Cytokine production by human monocyte-derived primary macrophages (hMDM) and co-incubated with live cells from the MCF series indicated along the X-axis, at 1:5 macrophages/cell ratio. This result is representative of two experiments with two independent donors. **(B)** THP-1 monocyte cell line-derived macrophages were co-incubated as described in **(A)**. Results are presented as mean values ± SEM of three experiments. **(C)** THP-1 macrophages were co-incubated in a transwell plate with cells plated on top of the transwell. Cytokines were assessed in a bead-based assay and normalized against the highest cytokine concentration observed in responses to one of the cell lines. Results are presented as mean values ± SEM of three experiments. Cytokine concentrations in co-incubations with MCFNeoT (NeoT), MCFT1 (T1) and MCFCA1 (CA1) were compared to MCF10A (10A) using Fisher LSD tests for each cytokine (represented by different color): *p<0.05, ****p<0.0001.

We incubated primary human monocyte derived macrophages (hMDM) for 24 hours with the MCF cell lines representing the various stages of malignant transformation, from normal (MCF10A) to premalignant (MCFNeoT and MCFT1) to invasive breast cancer (MCFCA1), and quantified 13 secreted cytokines using a cytometric bead array. We focused on i) cytokines with concentrations above the detection threshold, ii) cytokines secreted at different levels depending on the cancer cell line used in the co-incubation, iii) cytokines secreted in responses to more than one cell line, iv) cytokines for which concentrations were significantly different in co-culture compared to mono-culture. Following this screening method, we selected three cytokines, TNF-α, IL-1β and IL-10. We found that co-incubation of macrophages with MCF10A induced low levels of TNF-α and IL-1β and high levels of IL-10 (**Figure 1A**). IL-10 levels were lower in co-cultures with MCFNeoT, and there was no detectable TNF-α or IL-1β. TNF-α levels dramatically rose in co-incubation with MCFT1 and decreased but remained high with MCFCA1. While IL-1β paralleled TNF-α, increasing in co-cultures with MCFT1 and reaching even higher levels in response to MCFCA1, IL-10 levels continued to decrease reaching their lowest levels in co-cultures with MCFT1 and MCFCA1.

We repeated these experiments with THP-1 monocyte cell line-derived macrophages that allow high reproducibility and for which a recent study showed that they could be used as a simplified model of human macrophages even though the order of magnitude in cytokine secretion after polarization was lower in hMDM compared to THP-1 (20). We recapitulated the results from primary macrophages: decrease in IL-10 and increase in TNF-α and IL-1β occurred between MCFNeoT and MCFT1 (**Figure 1B**). The result was the same at 1:10 macrophage/MCF cell ratio (**Supplementary Figure S1A**). The production of these three cytokines by the MCF cell lines alone was low and could not account for the difference observed in co-incubation (**Supplementary Figure S1B**). We then tested the importance of cell-cell contact for establishing or maintaining the differences in cytokine patterns of expression between an earlier premalignant stage MCFNeoT and a later premalignant stage MCFT1. We performed the co-incubations in transwell plates where macrophages were seeded in the bottom section of the transwell, separated from the MCF cells that were plated in the top of the transwell (**Figure 1C**). The shift in cytokine expression patterns between MCFNeoT and MCFT1 was diminished in the absence of direct cell-cell contact, with higher TNF-α concentrations in response to MCF10A and MCFNeoT soluble factors and increased IL-10 levels in responses to MCFT1 and MCFCA1. IL-1β levels were not affected and remained higher in response to MCFT1 and MCFCA1 compared to MCF10A.

Compared to unstimulated macrophages, THP-1 in contact with MCF10A showed similar levels of TNF-α and IL-1β but higher levels of IL-10, suggesting that macrophages where inert when sensing MCF10A (**Supplementary Figure S2**). In contrast, when incubated with MCFT1 and MCFCA1, THP-1 macrophages secreted lower levels of IL-10 and a 2-fold increase in TNF-α and IL-1β compared to unstimulated macrophages, suggesting immune activation. In co-incubations with MCFNeoT, macrophages seemed to be in a transitional state with similar levels of IL-10 and TNF-α but higher levels of IL-1β than resting macrophages. Therefore, an activation threshold depending on cell-cell contact and sensing of surface molecules appeared to be between the two premalignant states, MCFNeoT representing benign hyperplasia and atypical hyperplasia represented by MCFT1 in both primary hMDM and THP-1 derived macrophages.

We were also able to identify a similar activation threshold in THP-1-derived dendritic cells (**Supplementary Figure S3**). In co-incubations with MCFT1 and MCFCA1, IL-1β concentration was increased whereas IL-10 concentration was decreased compared to co-incubation with MCF10A, similarly to what was observed in macrophages. TNF-α was however produced at very low levels by dendritic cells and instead IL-18, another proinflammatory cytokine primarily involved in polarized T-helper 1 was increased between MCFNeoT and MCFT1.

### Increase in Phagocytic Activity and Tumor Infiltration Coincides With the Macrophage Activation Threshold

**Figure 2:**
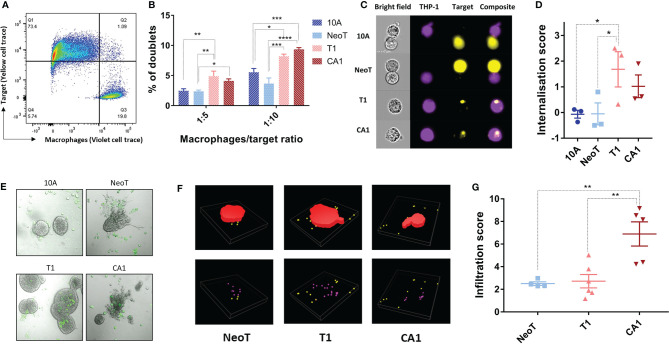
Defining thresholds for macrophage phagocytosis of MCF cell lines in suspension and infiltration into 3-D clusters. **(A)** Example of the gating strategy to identify macrophage-MCF cell doublets. Macrophages and MCFNeoT cells were labeled with Pacific Blue and PE cell tracers, respectively. Doublets (Q2) were identified as PacBlue, PE double positive cells. **(B)** Percentages of doublets in 1:5 and 1:10 macrophage/MCF cell ratio as indicated MCF10A (10A), MCFNeoT (NeoT), MCFT1 (T1) and MCFCA1 (CA1). Results are presented as mean values ± SEM of four experiments. Fisher LSD test: ****p<0.0001, ***p<0.001, **p<0.01, *p<0.05. **(C)** Examples of images taken on the AMNIS 24h after coincubation of macrophages and indicated cells (1:20 ratio). Cells were labeled as in B and gating was on live cells. **(D)** Internalization scores were calculated using the Internalization function in IDEAS 6.2. Results are presented as mean values ± SEM of three experiments. Fisher LSD test: *p<0.05**. (E)** Representative images of macrophages contacting MCF10A (10A) and MCFNeoT (NeoT); infiltrating MCFT1 (T1); destroying MCFCA1 (CA1) cell clusters. Macrophages were labeled with FITC (green) and added on top of cells in 3-D clusters grown in Matrigel. **(F)** Examples of 3-D reconstructions from z-stacked images using the NIS-Element software. 3-D clusters are represented in red, non-infiltrating macrophages in yellow and infiltrating macrophages in pink. **(G)** Infiltration score was calculated as the number of infiltrating macrophages divided by the cluster volume as described in Materials and Methods. Results are presented as mean values ± SEM of 4 clusters for MCFNeoT, 6 for MCFT1 and 5 for MCFCA1. Fisher LSD test: **p<0.01.

We wanted to know if the activation threshold that resulted in increased pro-inflammatory cytokine production extended to other macrophage functions. We used flow cytometry to examine the interaction of labeled macrophages (CellTrace Violet, **Figure 2A**, Q3) with the MCF cell lines (CellTrace Yellow, **Figure 2A**, Q1), and quantified the percentage of the total macrophage population that had formed doublets with the MCF cells (**Figure 2A**, Q2). Compared to MCF10A and MCFNeoT, macrophage incubation with MCFT1 and MCFCA1 yielded significantly higher number of doublets, in both 1:5 and 1:10 macrophage/MCF cell ratio (**Figure 2B**). Because these doublets can represent either phagocytosed cells or macrophage-MCF cell conjugates, we used the AMNIS instrument to visualize cell internalization. Indeed, the overlay of the fluorescent cell images allowed visualization of the MCF cells inside the macrophages distinguishing them from cell-cell conjugates (**Figure 2C**). We then used an internalization wizard and found that the doublets were mostly conjugates with MCF10A and MCFNeoT (low internalization score) whereas in the case of MCFT1, whole cells or cell debris were phagocytosed (high internalization score) (**Figure 2D**). Thus, we verified that the phagocytosis threshold was between MCFNeoT and MCFT1, matching the threshold for pro-inflammatory cytokine production.

To represent better the conditions of macrophage interaction with mammary epithelial cells during breast cancer progression *in vivo*, we cultured MCF cell lines in Matrigel for 7-10 days allowing them to form 3-D clusters, followed by the addition of macrophages and incubation for an additional 24h. A substantial number of labeled macrophages (CellTrace CFSE) migrated into the Matrigel and adhered to the 3-D clusters of all four cell lines (**Figure 2E**). MCFCA1 clusters, but not the other cell lines, appeared to be also damaged by macrophages (**Figure 2E**, CA1 panel). We then wanted to determine if the macrophages infiltrated the clusters and if that varied between the cell lines. After 3-D reconstruction (**Figure 2F**), we quantified the number of macrophages inside each cluster and determined an infiltration score normalized by the volume of the cluster. We found that macrophages adhered but did not infiltrate MCF10A. MCFNeoT and MCFT1 clusters were infiltrated similarly by macrophages, however, MCFCA1 clusters showed significantly higher infiltration scores (**Figure 2G**). We therefore observed a two-step infiltration threshold, with the first threshold between MCF10A and MCFNeoT when macrophages start to actively infiltrate the clusters and a second threshold between MCFT1 and MCFCA1 where macrophages infiltrate clusters at greater numbers. While the internationalization score was similar for the two cell lines, the greater infiltration in MCFCA1 clusters could explain the multiple observations of cluster destructions (**Figure 2E**).

### Querying Well-Known Molecules Involved in Malignant Transformation, Macrophage Activation and Phagocytosis as Potential Danger Signals at the Macrophage Activation Threshold

**Figure 3:**
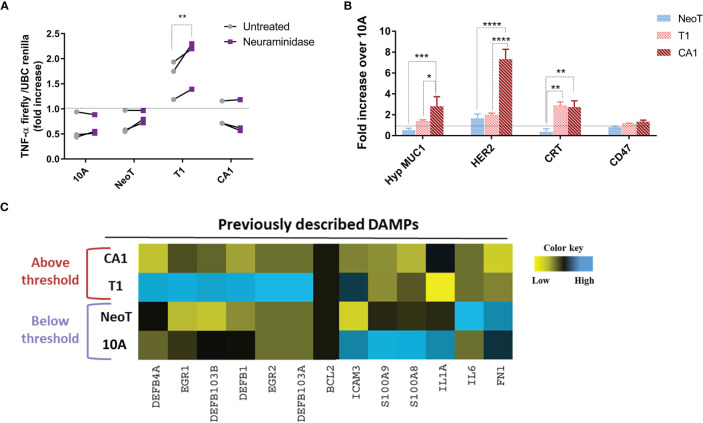
Querying known DAMPs as candidate danger signals for determining macrophage activation threshold in response to transformed cells. **(A)** Sialic acid removal by neuraminidase treatment of MCF cells, indicated along the X-axis, does not affect the threshold. Y-axis shows fold increase of TNF-α in THP-1 B5 macrophages after 24h incubation at 1:5 macrophage/cell ratio. Co-incubation with untreated cells is in gray and with neuraminidase-treated cells in purple. Results are representative of three experiments. Fisher LSD test: **p<0.01. **(B)** Differential expression of well-known tumor-associated molecules in indicated cells compared to MCF10A (10A, dashed line), assessed by flow cytometry. Results are presented as mean values ± SEM of three experiments. Fisher LSD test: ****p<0.0001, ***p<0.001, **p<0.01, *p<0.05. **(C)** Heatmap representation of expression of genes coding for previously described DAMPs. The color key was provided by the software and shows a gradient from low (log2 FC <–1) to high (log2 FC>1) expression.

As cell-cell contact was necessary to reveal the macrophage activation threshold, we focused our efforts on identifying cell surface molecules that could act as danger signals in malignant transformation. A common mechanism in tumor progression and metastasis is an alteration of glycosylation and sialylation (21). We investigated potential changes in the activation threshold after treating the MCF cells with neuraminidase, a glycoside hydrolase that removes sialic acids from the terminal positions of glycans and exposes the cryptic Tri/m-II, leading to an increased binding of calreticulin (CRT), the “eat me” signal, and phagocytosis (22). We found that neuraminidase treatment increased TNF-α concentration in responses to MCFT1 cells but did not move the threshold to the earlier premalignant stage or impact macrophage TNF-α response to the malignant MCFCA1 cells (**Figure 3A**).

Thus, we focused our next analyses on the expression of well-known tumor-associated antigens and damage-associated molecular patterns (DAMPs) to see which are associated with the transformation stages below *versus* above the macrophage activation threshold. We included in our analysis tumor antigens MUC1 and HER2, which have been reported to be over-expressed in breast cancer and to affect macrophage function (19, 23), and the “eat-me” signal CRT and the “don’t eat me” signal CD47 (24). We found that the hypoglycosylated form of MUC1 and Her-2/neu were significantly overexpressed on the surface of MCFCA1 compared to MCFNeoT but not on MCFT1, suggesting that they were not involved in setting the macrophage activation threshold (**Figure 3B**). In contrast, MCFT1 and MCFCA1 expressed significantly higher levels of CRT which may contribute to enhanced phagocytosis of these cells. All three cell lines expressed similar levels of CD47. Finally, we analyzed mRNA expression of a panel of DAMPs such as BCL2, EGR, ICAM-3, IL-1α, IL-6, defensins, fibronectin 1 and the S100 protein (25), and found no correlation of their expression with the macrophage activation threshold (**Figure 3C**). Nevertheless, MCFT1 showed a specific pattern of defensin and EGR gene expression compared to MCFCA1 that could contribute as additional signals for macrophage activation.

### Unbiased Identification of Potential New Candidates Acting as Danger Signals Associated With the Macrophage Activation Threshold

**Figure 4:**
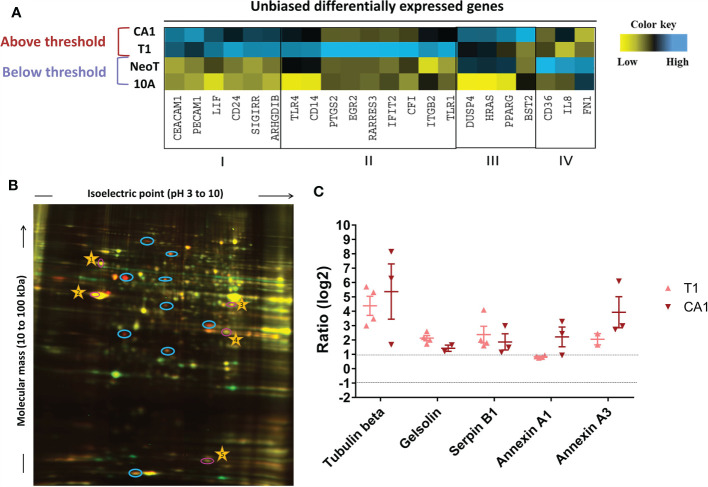
Unbiased identification of genes and proteins associated with the macrophage activation threshold. **(A)** Heatmap representation of expression of genes with a fold increase greater than 5 over MCF10A (10A). The color key was provided by the software and shows an expression gradient from low (log2 FC <–1) to high (log2 FC>1). I, genes upregulated in MCFT1 (T1) and MCFCA1 (CA1); II, genes upregulated in MCFT1(T1) only; III, genes upregulated in MCFCA1 (CA1) only; IV, genes upregulated in MCFNeoT (NeoT) only. **(B)** Differentially expressed proteins between MCF10A labeled with Cy3-NHS (green) and MCFT1 labeled with Cy5-NHS (red); labeled proteins were mixed and resolved on 2D-DIGE as described in Materials and Methods. Shared proteins migrate identically and appear as yellow spots. Blue circles mark spots unique to MCFT1 that were picked for sequencing. Numbered yellow stars were used in the quantification analysis as guiding spots. **(C)** Changes in expression of 2D-DIGE-identified proteins in MCFT1 and MCFCA1 relative to MCF10A. Results are presented as mean values ± SEM of 4 technical replicates.

We next took an unbiased approach to identify candidate danger molecules associated with macrophage activation. We first profiled the four MCF cell lines, quantifying expression of 579 immune response genes using the Nanostring nCounter Human Immunology V2 Panel. We identified 93 genes in MCFNeoT, 192 in MCFT1 and 136 in MCFCA1 that had log2 fold change of expression > 1 over MCF10A. When considering genes with a fold change of expression >5, we could identify four distinct groups of genes associated with various stages of malignant transformation: I) genes overexpressed in both MCFT1 and MCFCA1; II) genes overexpressed in MCFT1 only; III) genes overexpressed in MCFCA1 only; and IV) genes overexpressed in MCFNeoT only (**Figure 4A**). Of greatest interest are the genes in group I because they appear in MCFT1 and persist in MCFCA1, corresponding to the macrophage activation that starts in response to MCFT1 and continues in response to MCFCA1.

Because change in gene expression does not always translate to change in protein expression, we compared the proteome of each transformed cell line with the proteome of MCF10A and identified molecules specifically over-expressed in the premalignant and malignant cells. We extracted proteins from the monolayer cultures and labeled them with two different Cyanine-based, amine-reactive, minimal-labeling dyes and resolved them by 2D-DIGE (17) as described in Materials and Methods. **Figure 4B** is a representative 2D gel where proteins from MCF10A (red) and MCFT1 (green) were resolved and visualized as spots. We quantified the difference in expression of each protein by analyzing the pixel intensity of each spot in the images of 2D gels with Source Extractor across 2 biological and 4 technical replicates (**Supplementary Figure S4A**). After normalization that accounts for differences in dye intensities, we considered that proteins were significantly differentially expressed when they had a log2 fold change of spot intensity > 1 compared to MCF10A (**Supplementary Figure S4B**). Those protein spots were excised from the gel, digested into peptides with trypsin and subjected to mass spectrometry analysis. We found that five proteins were consistently over-expressed in MCFT1 and MCFCA1 but not in MCF10A and MCFNeoT: Tubulin beta, Gelsolin, Annexin A1, Annexin A3 and Serpin B1 (**Figure 4C**).

### The Candidate Danger Molecules Annexin A1 and CEACAM1 Participate in the Macrophage Activation Threshold

From the 11 candidate molecules identified with NanoString and 2D-DIGE methods, we decided to focus on 4 of them to further explore their surface expression and their impact on macrophage activation. We eliminated soluble factors (LIF) and proteins with structural or post-transcriptional changes (Tubulin beta) to focus on proteins for which antibodies were commercially available and their association with epithelial cancer progression had already been documented.

**Figure 5:**
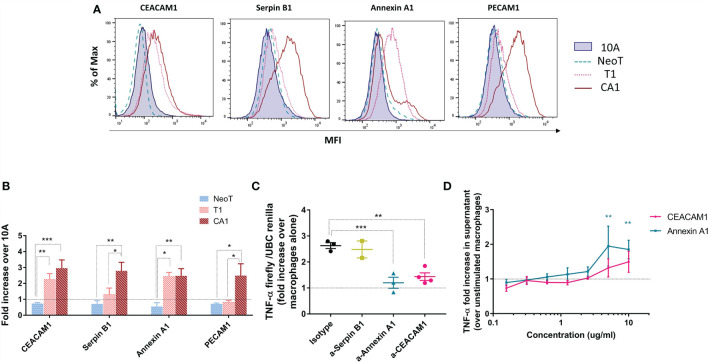
Candidate danger molecules differentially expressed above and below the macrophage activation threshold that promote inflammatory function. **(A)** Representative flow plots of CEACAM1, Serpin B1, Annexin A1 and PECAM1 expression in MCF10A (10A), MCFNeoT (NeoT), MCFT1(T1) and MCFCA1 (CA1). **(B)** Differential expression of selected cell surface proteins by MCFNeoT (NeoT), MCFT1 (T1) and MCFCA1 (CA1) relative to MCF10A (10A, dashed line), assessed by flow cytometry. Results are presented as mean values ± SEM of three experiments. Fisher LSD test: ***p<0.001, **p<0.01. **(C)** Fold increase of TNF-α expression in macrophages co-incubated for 24h at a ratio of 1 macrophage to 5 MCFT1 cells, preincubated for 30 min with either an isotype control antibody or antibodies against Serpin B1 (yellow), Annexin A1 (blue) and CEACAM1 (pink). Results are presented as mean values ± SEM of two to four experiments. Fisher LSD test: **p<0.01, *p<0.05. **(D)** Fold increase in TNF-α production in macrophages stimulated with different concentrations of Annexin A1 and CEA proteins for 24h compared to unstimulated macrophages, as assessed by ELISA. Results are presented as mean values ± SEM of three experiments. Fisher LSD test: **p<0.01.

We measured protein expression on the surface of the transformed cell lines by flow cytometry and expressed the results relative to MCF10A (**Figure 5A**). Serpin B1 and PECAM1 were significantly more highly expressed on MCFCA1 compared to MCF10A, while MCFT1 and MCFCA1 both expressed significantly higher levels of CEACAM1 and Annexin A1 (**Figure 5B**). This confirmed that the transcriptomic and proteomic differences observed between the cell lines were associated with differences in surface expression. The only discordance between our 2D-DIGE and flow cytometry analysis was in the case of Serpin B1, which is largely localized to the cytoplasm.

We attempted to interfere with the macrophage threshold by pre-treating MCF cell lines for 30 minutes with antibodies against these three molecules in order to block their recognition by macrophages. **Figure 5C** shows that antibodies against Annexin A1 and CEACAM1, but not against Serpin B1, lowered TNF-α production in response to MCFT1 to levels comparable to what we see in response to MCF10A and MCFNeoT (**Supplementary Figure S5A**). This effect was antibody-dose dependent (**Supplementary Figure S5B**). Finally, we confirmed the potential of our candidates to activate macrophages by stimulating THP-1 macrophages with CEACAM1 and Annexin A1 individually at different concentration (**Figure 5D**). We found that both proteins activate a TNF-α responses in a dose-dependent manner. Activation by Annexin A1 was significant at concentration above 2.5ug/ml and while CEACAM1 followed the same trends it did not reach significance.

### Evidence of a Threshold in Annexin A1 and CEACAM1 Expression in Human Breast Cancer Associated With Macrophage Activation

**Figure 6:**
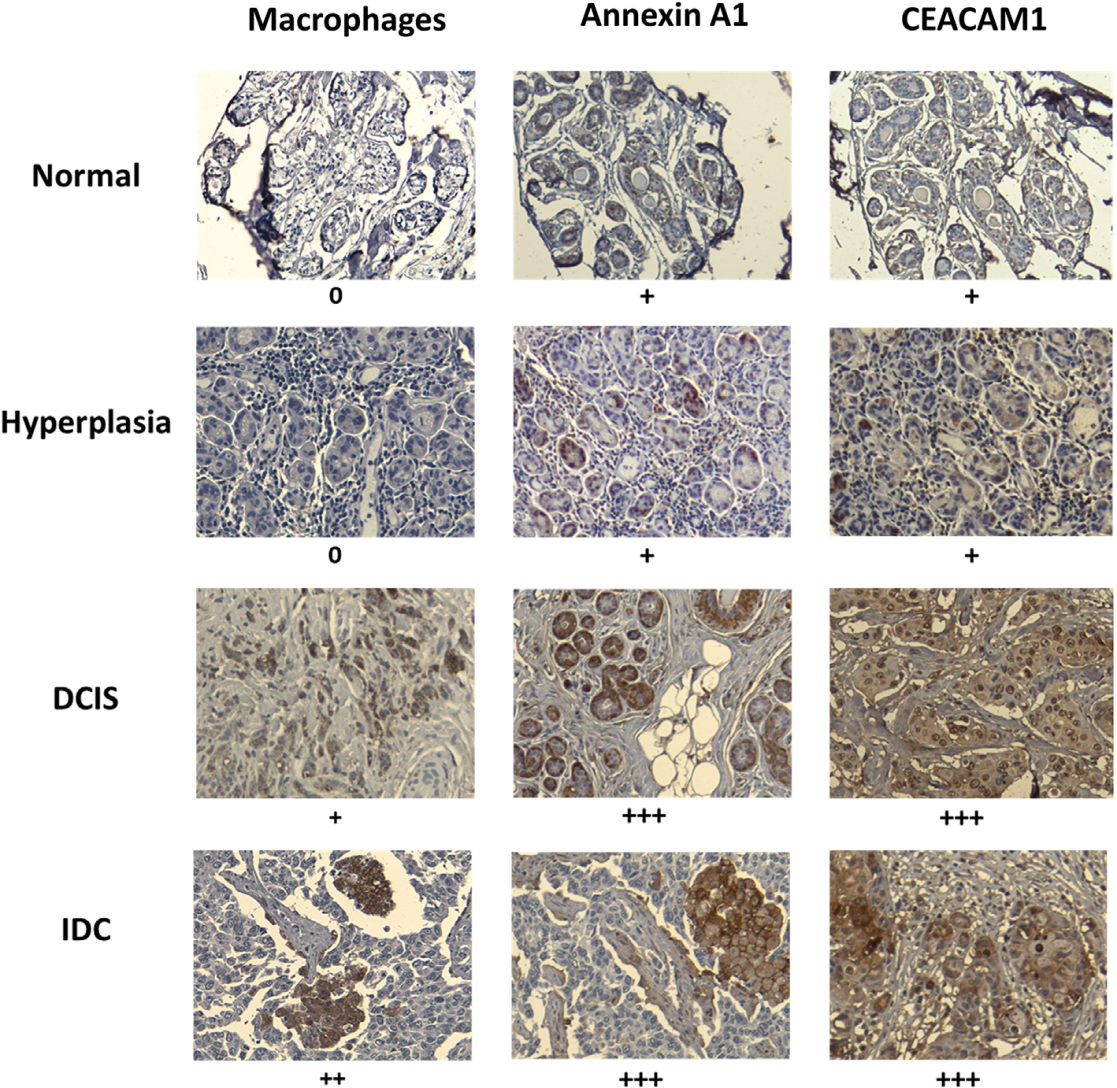
Macrophage infiltration and expression of Annexin A1 and CEACAM1 in human breast tissues at various stages of transformation mirror the *in vitro* observed activation threshold. Example of paraffin-embedded samples of breast tissues, normal (1 of 8 total), hyperplasia (1 of 5 total), ductal carcinoma *in situ* (DCIS) (1 of 8 total) and invasive ductal carcinoma (IDC) (1 of 6 total), sectioned and stained with relevant antibodies (see *Materials and Methods*). Slides were scanned at 10X magnification in order to select for a high-resolution image at 20X. Images were scored by measuring the percentage of IHC positively labeled cells: +, <25%; ++, 25 to 50%; +++, 50 to 75%; and ++++ > 75%. Representative images are shown. The “+” signs refer to results from all analyzed samples in each tissue type.

We examined human breast tissue samples of normal breast ducts (MCF10A-like), preneoplastic hyperplasia (MCFNeoT-like), ductal carcinoma *in situ* (DCIS, MCFT1-like) and invasive ductal carcinoma (MCFCA1-like), for evidence of a macrophage infiltration threshold, which we detected by the intensity of staining for the macrophage marker CD68. We saw no macrophages in the normal and in hyperplastic tissue sections (**Figure 6**). The first evidence of macrophage infiltration was found at the DCIS stage and it increased greatly in invasive ductal carcinoma (IDC). We also looked for differential expression of the candidate danger molecules Annexin A1 and CEACAM1 and saw, consistent with our cell line data, increased Annexin A1 and CEACAM1 expression beginning at the DCIS (MCFT1-like) stage and continuing in invasive cancer.

## Discussion

Macrophages are known to be important effectors of cancer immunosurveillance (26) through programmed cell removal and activation of TLR pathways (27). However, because cancer cells closely resemble normal cells it is still unknown how early in tumor development can macrophages sense and eliminate abnormal cells or initiate adaptive immunity against them. The results described here give the first evidence in an *in vitro* model system of a threshold of activation and phagocytosis that is observed in macrophages that interact with cells at various stages of malignant transformation. We were able to detect such a threshold by co-culturing macrophages with a series of cell lines that were developed to recapitulate the progression of breast cancer (15). We were able to show a switch from low baseline levels of TNF-α production in response to normal MCF10A to high levels in response to atypical hyperplasia, a premalignant stage of disease. This activation of TNF-α production was dependent on cell-cell contact and was contemporaneous with an increase in phagocytic activity of macrophages against that same premalignant stage. The difference observed between MCFT1 and MCFCA1 in terms of 3-D cluster infiltration could be explained by the difference in “find-me” signal secretion (28). However, we demonstrated that *in vivo*, the threshold for infiltration of macrophages in malignant lesions was similar to the activation threshold, suggesting that our 3-D culture system might not reproduce all the interactions in the tumor microenvironment, especially those with other immune cells. Similarly, our system did not allow to measure the consequences of long-term interactions between tumor cells and macrophages. Indeed, tumor cells can promote a pro-tumoral phenotype in macrophages, which in turn stimulate angiogenesis and enhance tumor cell invasion and motility. Future studies will explore the long-term interactions between early premalignant lesions and macrophages and how they impact their anti-tumoral activity.

Macrophages have been shown to distinguish cancer cells from normal cells by the DAMPs they express on their surface. DAMPs are recognized by TLR receptors on macrophages and trigger a molecular cascade leading to pro-inflammatory responses. This does not appear to be the main mechanism in the setting of premalignancy. We did not find an association between the expression of previously described DAMPs or known breast tumor-associated antigens with the threshold of activation of premalignant cells. Rather our study showed the importance of other molecules still poorly investigated for their role as “danger signals” or DAMPs, that appear to distinguish “self” from pre-cancer. Among those we found that we could block Annexin A1 and CEACAM1 with antibodies and abrogate the threshold for macrophage activation. They were also individually sufficient to activate TNF-α production by macrophage as previously described for CEA (29). We confirmed that they are overexpressed *in vivo* as early as ductal carcinoma *in situ*, which is represented by the premalignant MCFT1 cell line. In the Human Protein Atlas, expression of Annexin A1 (p=0.025) and CEACAM1 (p=0.007) was associated with an increased survival in breast cancer patients. Annexin A1 is an immune-modulating protein with diverse functions, one of which is an “eat me signal,” that plays multiple roles in cancer growth and metastasis (30). Annexin A1 binds to the formyl peptide receptor (FPR) 2, a pathogen recognition receptor that triggers immune responses (31). CEACAM1 is well-known as a tumor-associated antigen over-expressed primarily in colorectal cancers but also in breast cancer (32), and it has been shown to activate inflammatory responses and promote differentiation of human macrophages (29, 33). Macrophages expressed two receptors for CEA molecules, TIM3 that has been identified as a receptor of CEACAM1 on innate cells, and the heterogeneous nuclear ribonucleoprotein M (CEAR) that is involved in immune activation signaling (34, 35).

The origin of ancestral myeloid phagocytes is linked to the appearance of vertebrates 300 million years ago (36) and phagocytic activity was also identified in invertebrate such as starfishes (37). Cancer probably appeared long before that with the transition to multicellularity more than half a billion years ago (38). Therefore, macrophages have been under strong selective pressure to eliminate aberrant cells in the absence of adaptive effectors appearing in mammals. Our study supports that macrophages are involved in the recognition of developing cancer with a threshold of activation with advanced premalignant stages based on highly conserved danger signals. Indeed, Annexin A1 is expressed from mammals to birds with a remarkable conservation of the intron-exon organization (39, 40), while the CEACAM1 gene family is evolving more rapidly but several orthologous genes can be found in distantly related mammals (41). This rather late activation threshold might reflect a trade-off between immunosurveillance and auto-immunity. In fact, because cancer is mainly a post-reproductive disease (42), natural selection is likely to strongly select mechanisms that increase survival in early life such as the ones that are limiting auto-immune responses even at the detriment of letting early premalignant lesions grow. In addition, the evolution of immunosurveillance has probably faced another trade-off related to inflammation associated with cell destruction that can potentially lead to *de novo* damages in surrounding normal cells and tumorigenesis, a situation that has been envisioned in responses to immune-checkpoint inhibitors (43). In light of these constraints, the evolution of inflammatory responses to only fitness-decreasing phenotypes of cancer (i.e., clinically malignant) seems to represent a beneficial equilibrium. However, recent evidence suggests that this equilibrium, and therefore the threshold of activation, might change depending on the importance of particular cells and organs for survival of the individual (44, 45). The eradication of non-essential cells (such as melanocytes) is affordable to the organism and therefore malignant transformation could trigger macrophage activation earlier than what we observed in the breast. Further studies should explore the existence and characteristics of the innate immune activation threshold in different cancer types.

The data emerging from our study suggest significant opportunities to use the “danger signals” on premalignant lesions to develop novel targeted chemoprevention and immunoprophylactic strategies. Drugs could also be developed to lower the discrimination threshold and therefore eliminate more efficiently earlier stages of premalignancy. However, those drugs will have to be carefully assessed for associated side-effects as early premalignant stages still share a lot of similarities with normal cells. Tampering with the immune tolerance trade-off could have dangerous auto-immune consequences and thus identification of markers that are present in premalignant lesions but absent or low in normal cells is crucial for the development of safe drugs. In addition to supporting cancer prevention, a better understanding of mechanisms selected by evolution for a decreased tolerance of the immune system to premalignant cells could have implications for the management of auto-immune disorders.

## Data Availability Statement

The datasets presented in this study can be found in online repositories. The names of the repository/repositories and accession number(s) can be found below: The Gene expression data have been deposited and can be found with the accession number GSE181585 at: https://www.ncbi.nlm.nih.gov/geo/query/acc.cgi?acc=GSE181585. Similarly, the mass spectrometry proteomics data have been deposited to the ProteomeXchange Consortium *via* the PRIDE partner repository with the dataset identifier PXD027746.

## Author Contributions

CJ, RG, and OF contributed to conception and design of the study. CJ and MD conducted the *in vitro* experiments. CJ, SB, and JM performed the 2D DIGE analyses. CJ wrote the first draft of the manuscript. RG, JM, and OF wrote sections of the manuscript. All authors contributed to manuscript revision, read, and approved the submitted version.

## Funding

This work was supported by NIH grant R35 CA210039 to OJF and Forbeck Foundation grant to CJ.

## Conflict of Interest

OF is on the External Advisory Boards of GeoVax, Biovelocita, Immodulon, IASO and PDS Biotech.

The remaining authors declare that the research was conducted in the absence of any commercial or financial relationships that could be construed as a potential conflict of interest.

## Publisher’s Note

All claims expressed in this article are solely those of the authors and do not necessarily represent those of their affiliated organizations, or those of the publisher, the editors and the reviewers. Any product that may be evaluated in this article, or claim that may be made by its manufacturer, is not guaranteed or endorsed by the publisher.
