# Supplementary material for: Identification of Cell Surface Molecules That Determine the Macrophage Activation Threshold Associated With an Early Stage of Malignant Transformation
Source: Front Immunol. 2021 Oct 12;12:749597. doi: 10.3389/fimmu.2021.749597 (PMC8546176; doi:10.3389/fimmu.2021.749597)
Supplement: Supplementary file 1 [file DataSheet_1.docx]

**Supplementary materials**

**A**

**
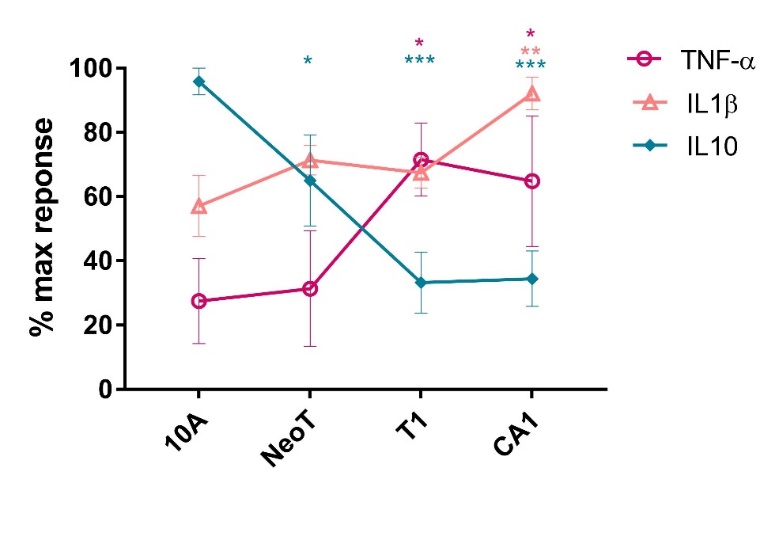
**

**B**


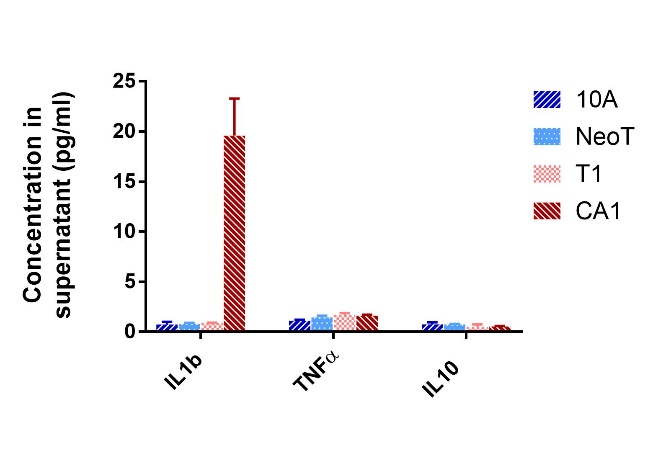


**Figure S1:** **A)** Macrophages were co-incubated with live target cells to 1:10 macrophages/target ratio. Results are presented as mean values ± SEM of three experiments. **B)** MCF cell lines were incubated for 24 hours before supernatant collection. The secretion of cytokines was assessed by a cytometric-based assay. Results are presented as mean values ± SEM of two technical replicates and representative of three biological replicates.


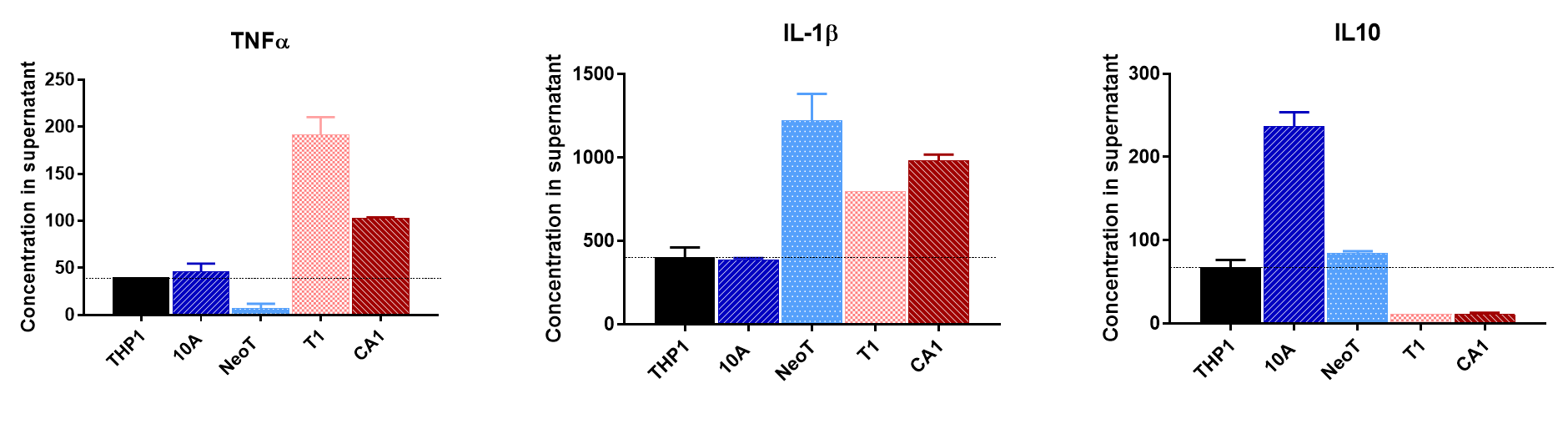


**Figure S2:** **Macrophage cytokine secretion in response to different steps of breast cancer progression.** Macrophages were co-incubated with the different MCF cell lines. The secretion of cytokines was assessed by a cytometric-based assay. Results are presented as mean values ± SEM of two technical replicates and representative of three biological replicates.

**
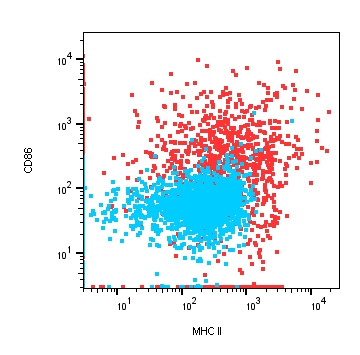
**
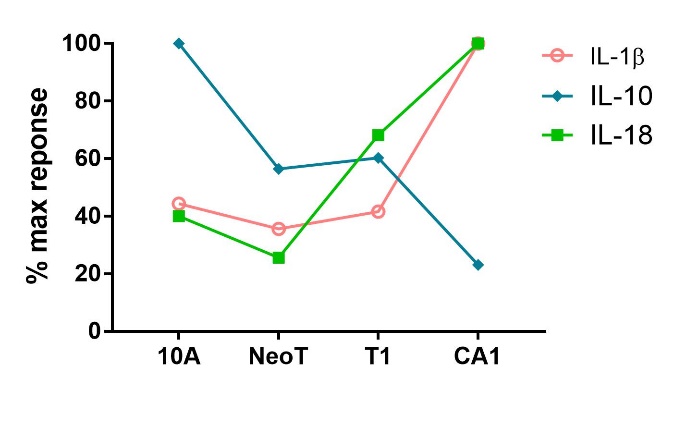


**A**

**B**

**Figure S3: Characterization of the dendritic cell cytokine responses to different steps of breast cancer progression.** **A**) Expression of the DC markers CD86 and MHC II in THP-1 (blue) and DC (red) generated by growing THP-1 monocytes in 200 ng/ml of rhIL-4, 100 ng/ml rhGM-CSF, 20ng/ml rhTNF-α and 200ng/ml ionomycin. **B)** After 24h of co-incubation, the secretion of cytokines was assessed by a cytokine-based assay and normalized against the highest cytokine concentration observed in responses to one of the cell lines. Cytokine concentrations in co-incubation with MCFNeoT, MCFT1 and MCFCA1 were compared to MCF10A co-incubation with Dunnett’s multiple comparisons test for each cytokines (represented by different color): ***p<0.001, **p<0.01, *p<0.05.


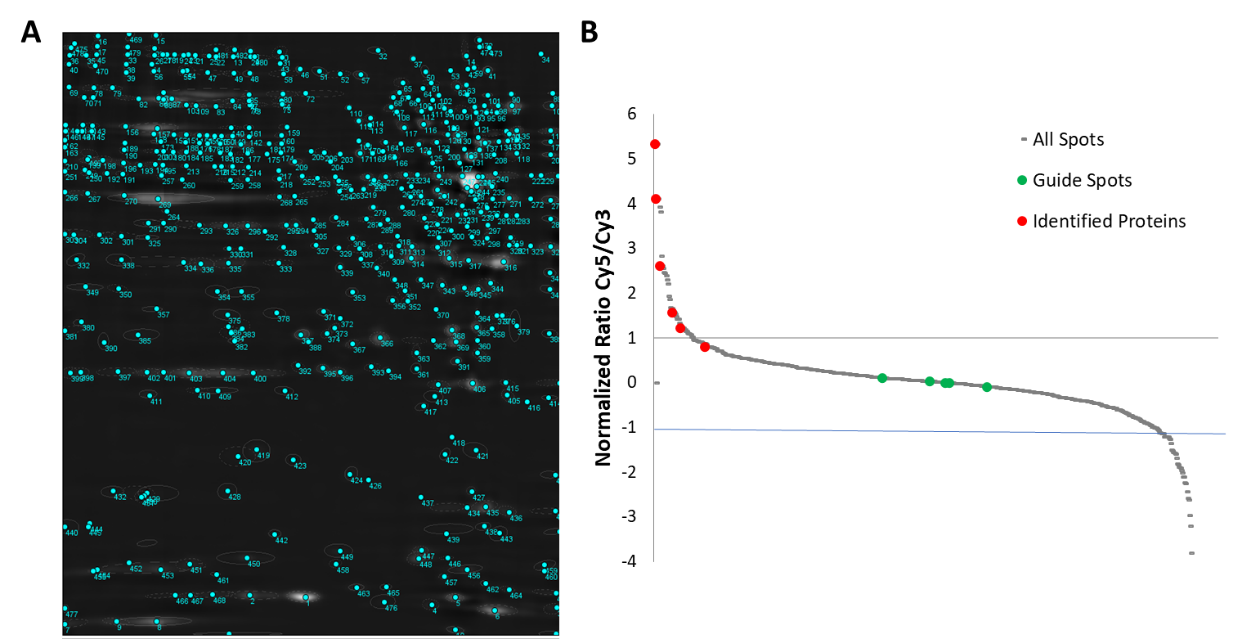


**Figure S4:** **Quantification of spot intensity in 2D gels**. **A)** Representation of the results of the quantification analysis using Source Extractor, the pixel intensity of each blue circles and numbers were extracted and Cy3/Cy5 ratios were calculated for each of them. **B)** After normalization by the mean ratio of the guiding spots, spot intensities were log transformed. Spots with log2 ratio > 1 were identified as significantly over-expressed. Spots picked for sequencing are represented in red and guiding spots are represented in green.

**
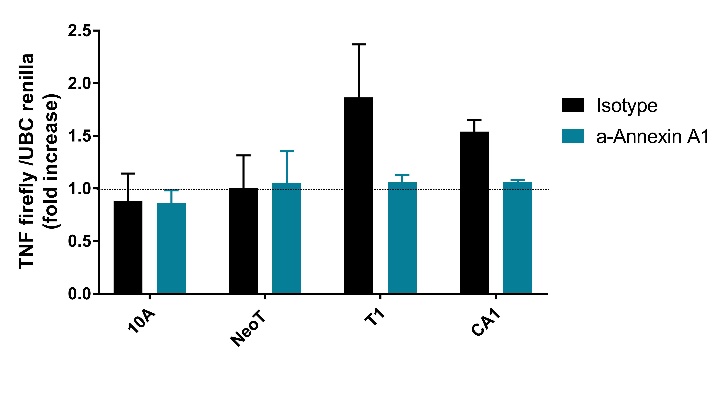
**
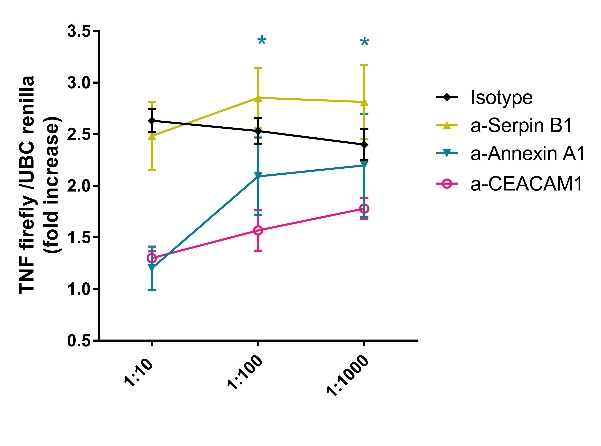


**B**

**A**

**Figure S5: (A)** Blocking of Annexin A1 in the four cell lines at 1:10 concentration. The experiments were conducted in the conditions described in Material and Methods. **(B)** Dose-dependent TNF-α responses with 1:10 to 1:1000 dilutions of blocking antibodies. Results are presented as mean values ± SEM of two to four experiments.
